# Supplementary material for: Rep15 interacts with several Rab GTPases and has a distinct fold for a Rab effector
Source: Nat Commun. 2022 Jul 23;13:4262. doi: 10.1038/s41467-022-31831-1 (PMC9308819; doi:10.1038/s41467-022-31831-1)
Supplement: Supplementary file 1 — Supplementary Information [file 41467_2022_31831_MOESM1_ESM.pdf]

Supplementary information for

Rep15 interacts with several Rab GTPases and has a distinct fold for a Rab effector

Rai *et al*.,

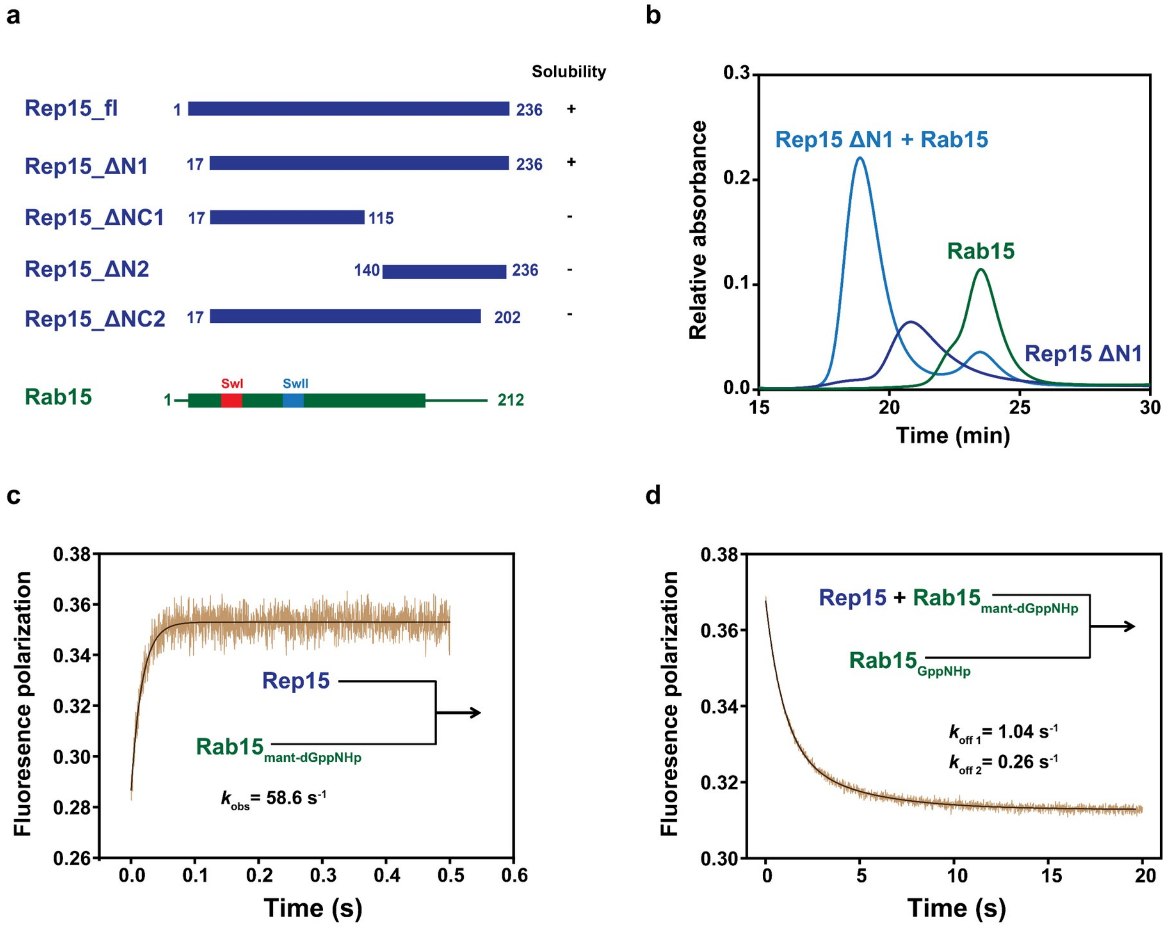

Supplementary Fig. 1: Biochemical characterization of Rep15:Rab15 interaction. **a** Schematic of full-length, several deletion constructs of Rep15 and full-length Rab15. Switch I and switch II regions of Rab15 are shown in red and sky blue, respectively. **b** Interaction of Rep15 ΔN1 with $Rab15_{GppNHp}$ was analyzed by analytical size exclusion chromatography. **c** Association of the Rep15 with $Rab15_{mant\text{-}dGppNHp}$ leads to an increase in fluorescence polarization and data is fitted to a single exponential. The change in fluorescence polarization of 1 μM $Rab15_{mant\text{-}dGppNHp}$ upon 5 μM Rep15 binding is shown. **d** Dissociation of Rab15 from the Rep15 was determined by monitoring the decrease of fluorescence polarization after mixing a complex of Rep15:$Rab15_{mant\text{-}dGppNHp}$ (2 μM) with a 25-fold excess of non-fluorescent $Rab15_{GppNHp}$ and data is fitted to a double exponential.

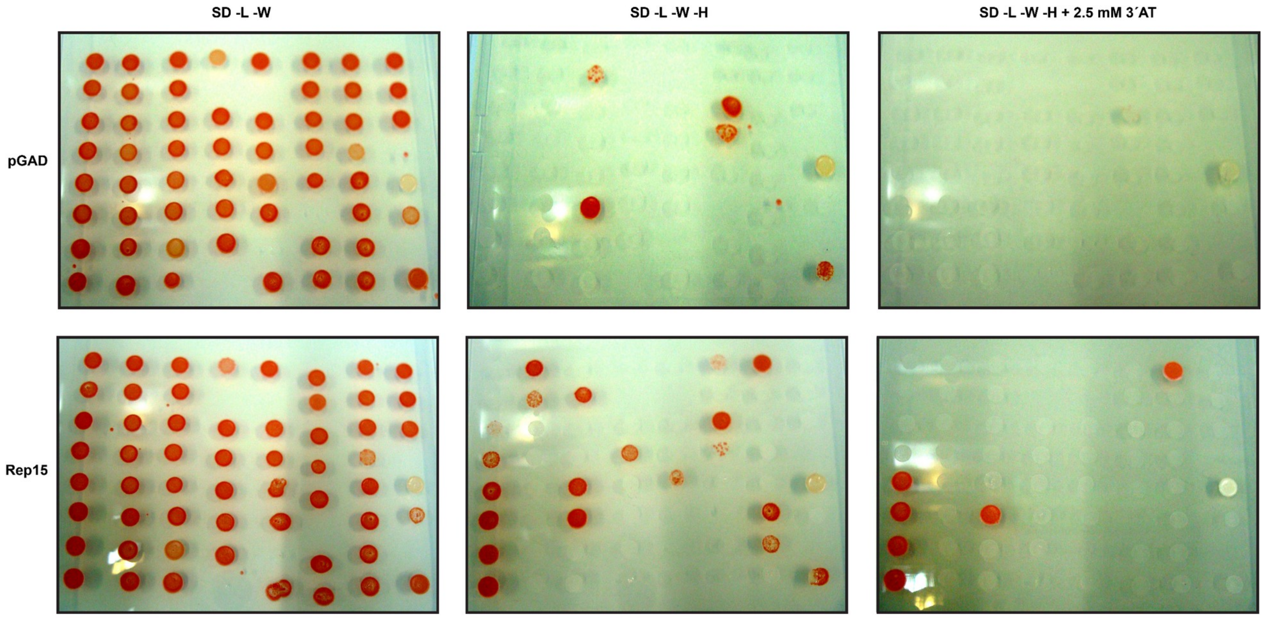

Rab1A	Rab4A	Rab7A	Rab11B	Rab20	Rab27B	Rab34	Rab40B
Rab1B	Rab4B	Rab7B			Rab28	Rab35	Rab40C
Rab2A	Rab5A	Rab8A	Rab13	Rab22A	Rab29	Rab36	Rab41
Rab2B	Rab5B	Rab8B	Rab14	Rab23	Rab30	Rab37	
Rab3A	Rab5C	Rab9A	Rab15	Rab24	Rab31	Rab38	+ve con
Rab3B	Rab6A	Rab9B	Rab17	Rab25		Rab39A	-ve con
Rab3C	Rab6B	Rab10	Rab18		Rab33A	Rab39B	
Rab3D	Rab6C	Rab11A		Rab27A	Rab33B	Rab40A	pLexA

Supplementary Fig. 2: A yeast two-hybrid screen between Rep15 (pGADT7-Rep15) and dominant active Rab (QL; pLexA) library. Cells were grown on SD-LW, SD-LWH and SD-LWH plus 2.5 mM 3-Aminotriazol (3'-AT), a competitive inhibitor of the HIS3 gene product. The experiment was also performed with the corresponding empty vector pGAD (pGADT7) to exclude auto-activation. Positive control: pGBKT7-53/pGADT7-T and negative control: pGBKT7-Lam/pGADT7-T. Y2H analysis identified interactions between the Rep15 and Rab3 paralogs/Rab34.

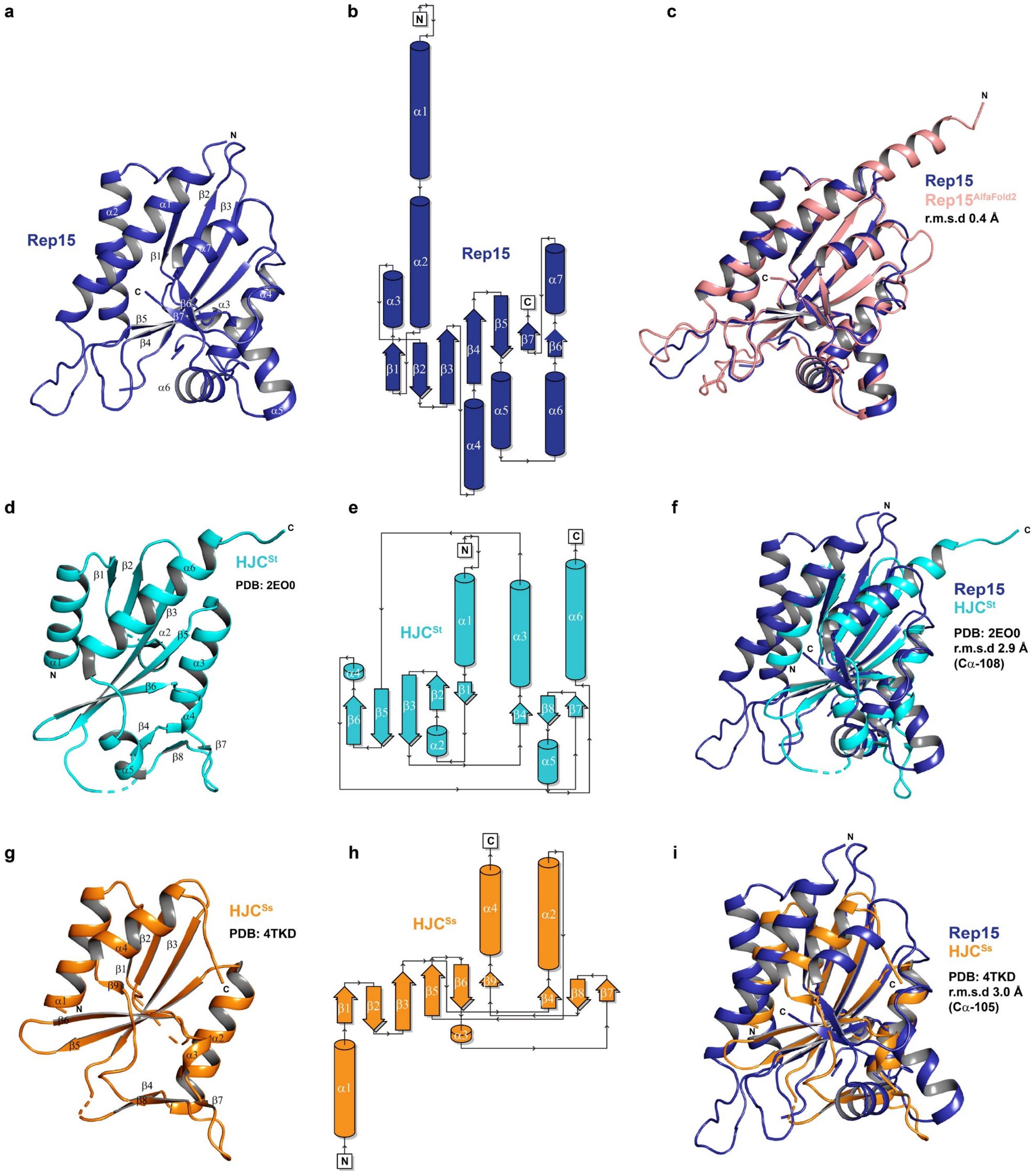

Supplementary Fig. 3: Topology of the Rep15. a-b Cartoon representation and topology diagram of human Rep15. **c** Structural overlay of Rep15 crystal structure (blue) with the model built by AlphaFold2[1] (salmon pink). **d-e** Cartoon representation and topology diagram denoting secondary structural elements of Holliday Junction Resolvase ST1444 from *Sulfurisphaera tokodaii* str. 7[2]. **f** Structural superposition of Rep15 (blue) and *Sulfurisphaera tokodaii* HJC (cyan, PDB: 2EO0). The topology diagrams were made with the PDBsum[3] webserver. **g-h** Cartoon representation and topology diagram denoting secondary structural elements of *Sulfolobus solfataricus* Holliday Junction Resolvase[4]. **i** Structural superposition of Rep15 (blue) and *Sulfolobus solfataricus* HJC (orange, PDB: 4TKD).

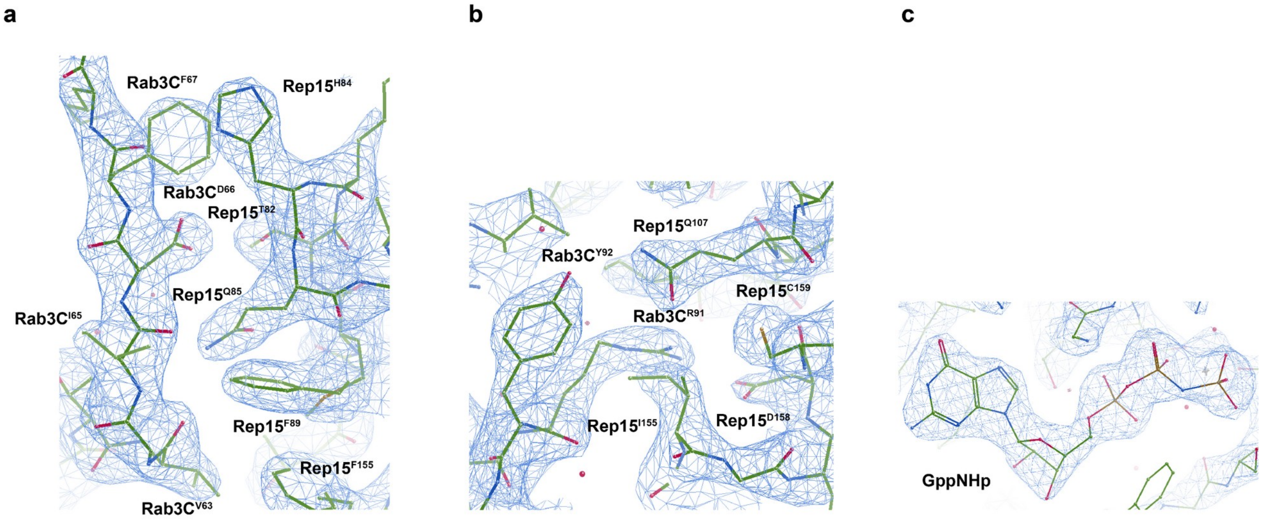

Supplementary Fig. 4: The 2Fo-Fc electron density map. a-b Rep15:Rab3C$_{GppNHp_Q222H_10\text{-}227}$ (bovine) binding interface, contoured at 1.5 σ. **c** GppNHp, contoured at 2.5 σ.

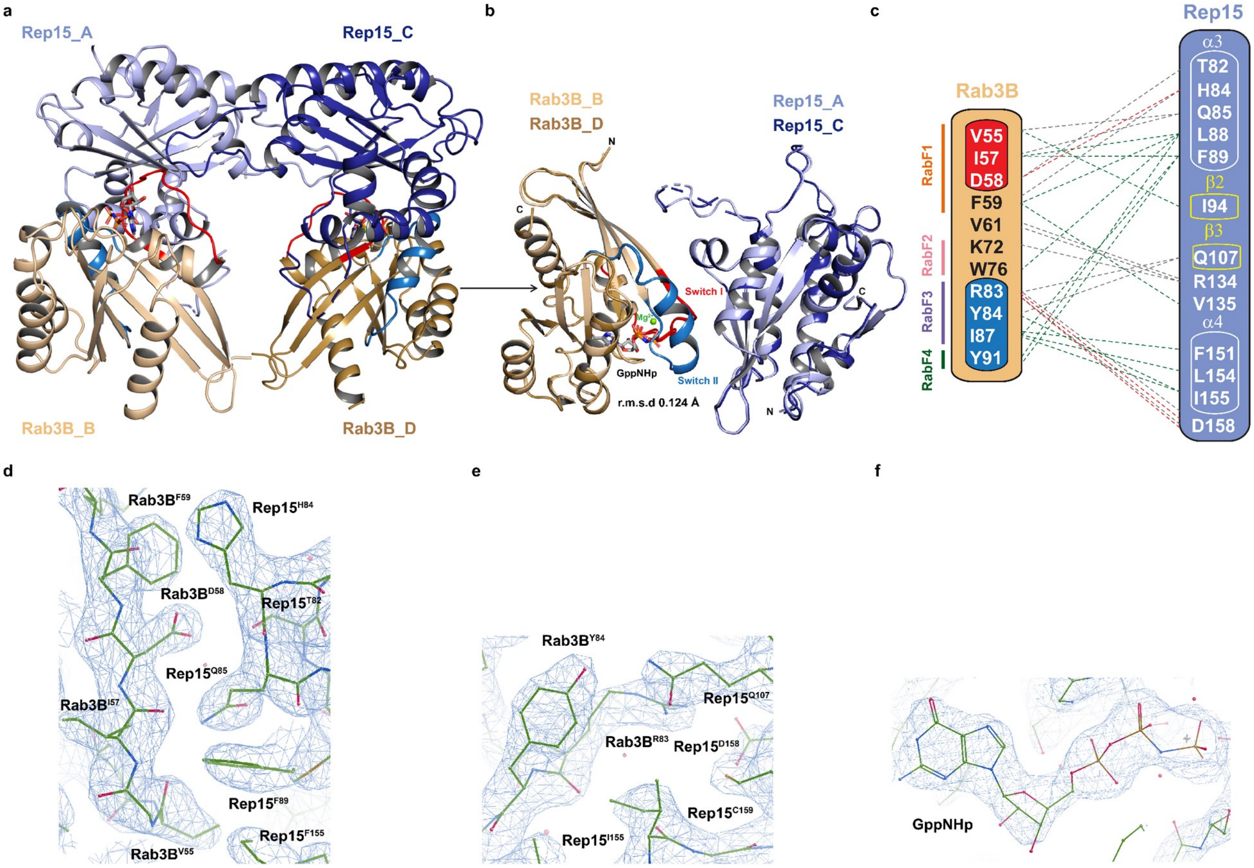

Supplementary Fig. 5: Crystal structure of the Rep15:Rab3B$_{GppNHp}$ complex. a-b Cartoon representation of the Rep15:Rab3B$_{GppNHp}$ complex. Rab3B (wheat^{chainB} and brown^{chainD}) binds to Rep15 (lavender^{chainA} and blue^{chainD}) via its switch I, switch II and interswitch regions. Switch I and switch II regions are highlighted in red and sky blue, respectively. Mg^{2+} ion is shown as a green sphere and GppNHp is shown in stick representation. **c** Schematic illustration of the Rep15:Rab3B$_{GppNHp}$ binding interface. Hydrogen bonds and ionic interactions are shown in gray and red dashed lines, respectively. Green dashed lines indicate the hydrophobic interactions. RabF1, RabF2, RabF3, and RabF4 motifs are shown in orange, pink, purple, and green, respectively. **d-f** The 2Fo-Fc electron density map. **d-e** Rep15:Rab3B binding interface, contoured at 1.5 σ. **f** GppNHp, contoured at 2.5 σ.

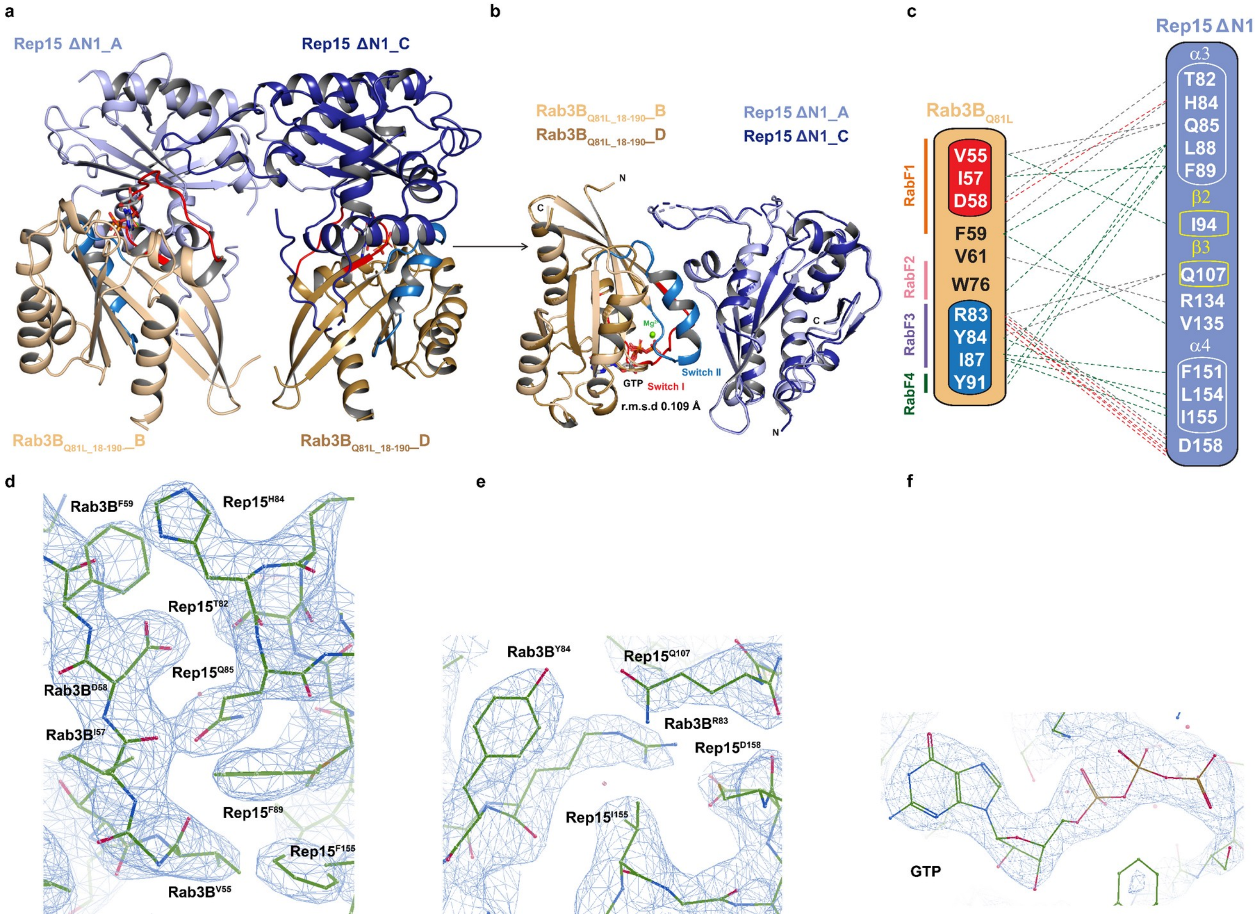

Supplementary Fig. 6: Crystal structure of the Rep15 ΔN1:Rab3B$_{Q81L_18\text{-}190}$ complex. a-b Cartoon representation of the Rep15 ΔN1:Rab3B$_{Q81L_18\text{-}190}$ complex. Rab3B$_{Q81L_18\text{-}190}$ (wheat^{chainB} and brown^{chainD}) binds to Rep15 ΔN1 (lavender^{chainA} and blue^{chainD}) via its switch I, switch II and interswitch regions. Switch I and switch II regions are highlighted in red and sky blue, respectively. Mg^{2+} ion is shown as a green sphere and GTP is shown in stick representation. **c** Schematic illustration of the Rep15 ΔN1:Rab3B$_{Q81L_18\text{-}190}$ binding interface. Hydrogen bonds and ionic interactions are shown in gray and red dashed lines, respectively. Green dashed lines indicate the hydrophobic interactions. RabF1, RabF2, RabF3, and RabF4 motifs are shown in orange, pink, purple, and green, respectively. **d-f** The 2Fo-Fc electron density map. **d-e** Rep15 ΔN1:Rab3B$_{Q81L_18\text{-}190}$ binding interface, contoured at 1.5 σ. **f** GTP, contoured at 2.5 σ.

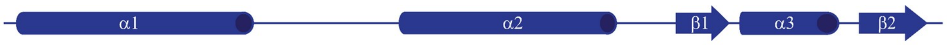


```
hRep15   MGQKASQQLALKDSKEVPVVCEVVSEAIVHAAQKLKEYLGFEYPPSKLCPAANTLNEIFLIHFITFCQEKGVDEWLTTTKMTKHQAFLFGADWIWTFWGS  100
mRep15   MGQKASQQVAPKDSQEVLAMCEVVSAAISHAAQKLKEYLGFEYPLSRLCLAASSLTEVFLVHFVTFCQERGADEWLTTTKMTKHQAWLFGADWIWTFWGP  100
sRep15   MGQKPSQQLALQDSEEVISISEVVSGAMVHAAQKLKEYLGFEDTLSHLCPAPNTLNEIFLIYFVTFCQEKGVDKWLTTTKMTKHQALLFGADWIWTFWGP  100
bRep15   MGQKPSQQLAPKD-KEVLNVCEVVSGAIIHAAQKLKAYLGFEDTLSNLCPDSNTLNEIFLIHFVTFCQEKGADKWLTTTKMTKHQAFLFGADWVWTFWGS  99
gRep15   MGQKASQQLALKDSKEVPVVCEVVSEAIVHAAQKLKEYLGFEYPPSKLCPAANTLNEIFLIHFITFCQEKGVDEWLTTTKMTKHQAFLFGADWIWTFWGS  100
cRep15   MGQKSSQQLPLKDSKEVSGVCEVVTEAIVHAAQKLKEYLGFEDPLSNLWPASNTLNEIFLIHFITYCQEKGVDEWLTTTKMTKHQAVLFGADWIWTFWGS  100
         **** ***:  :* :**  :.***: *: ******* *****   *.*    .:*.*:**::*:*:***:*.*:************ ******:*****
```

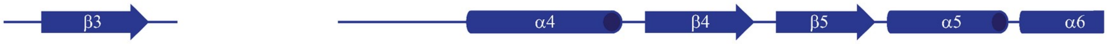


```
hRep15   DKQIKLQLAVQTLQMSSPPPVESKPCDLSNPESRVEESSWKKSRFDKLEEFCNLIGEDCLGLFIIFGMPGKPKDIRGVVLDSVKSQMVRSHLPGGKAVAQ  200
mRep15   DKQIRLQVAVQTLRMASLPLTDPKSCESR------GEESWKKGRFDKLQEFCNLVGEDCLGLFIIFGVPGEPKAIRGVVLESVRNGMMESQLPGRKAVEQ  194
sRep15   DKQIRLQLAVQTLQMSSLP-LESKPCDLSTPEARAEEPSSKTSRFDKLEEFCSLIGEDCLGLFIIFGVPGNPKDIRGVVLDSVKHEIASGHLPGGTAVAR  199
bRep15   DKQIQLQLAVQTLQMASLPPVESDP---SNPDSKAEKSFSKKSRFDKLEEFCNLIGEDCLGLCIIFGVPGNPKDIRGVVLDSVKRETTKGHLPGGTAVAR  196
gRep15   DKQIKLQLAVQTLQMSSPPPVESKPCDLSNPESRVEESSWKKSRFDKLEEFCNLIGEDCLGLFIIFGMPGKPKDIRGVVLDSVKSQMVRSHLPGGKAVAQ  200
cRep15   DKQIRLQLAVQTLQMSSLPAVESKPYNFSNLESKAEESSRKRSRFDKLEEFCNLIGEDCLGLFIIFGVPGKPKDIRGVVLDSVKSETVTGHLPGGKAVAQ  200
         ****:**:*****:*:* *  : .           :   *  .*****:***.*:******* ****:**:** ******:*:*:*::*: *:**: ***
```

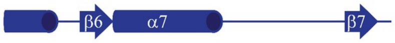


```
hRep15   FVLETEDCVFIKELLRNCLSKKDGLREVGKVYISIL 236
mRep15   FVLETKDCISIKELLGNCLSKRDGLSDMGRVYIRIL 230
sRep15   FILETEECVSIRELLGNCLSKKDGLREVGKAYISIL 235
bRep15   FILETEDCISIRELLGNCLSKKDGLREVGKVYISIL 232
gRep15   FVLETEDCVFIKELLRNCLSKKDGLREVGKVYISIL 236
cRep15   FVLDTEDCVSIRELIGNCLSKEDGLREMGKVYISIL 236
         *:*:*::*: *:**: *****.*** ::*:.** **
```

Supplementary Fig. 7: Sequence alignment of human Rep15 with mammalian homologs from *Mus musculus* (mRep15), *Sus scrofa* (sRep15), *Bos taurus* (bRep15), *Gorilla gorilla gorilla* (gRep15) and *Canis lupus familiaris* (cRep15) using Clustal Omega[5]. Identical residues are denoted by *, strongly similar residues by : and . indicates weakly similar residues. Residues involved in Rab3C interactions are labeled in red.

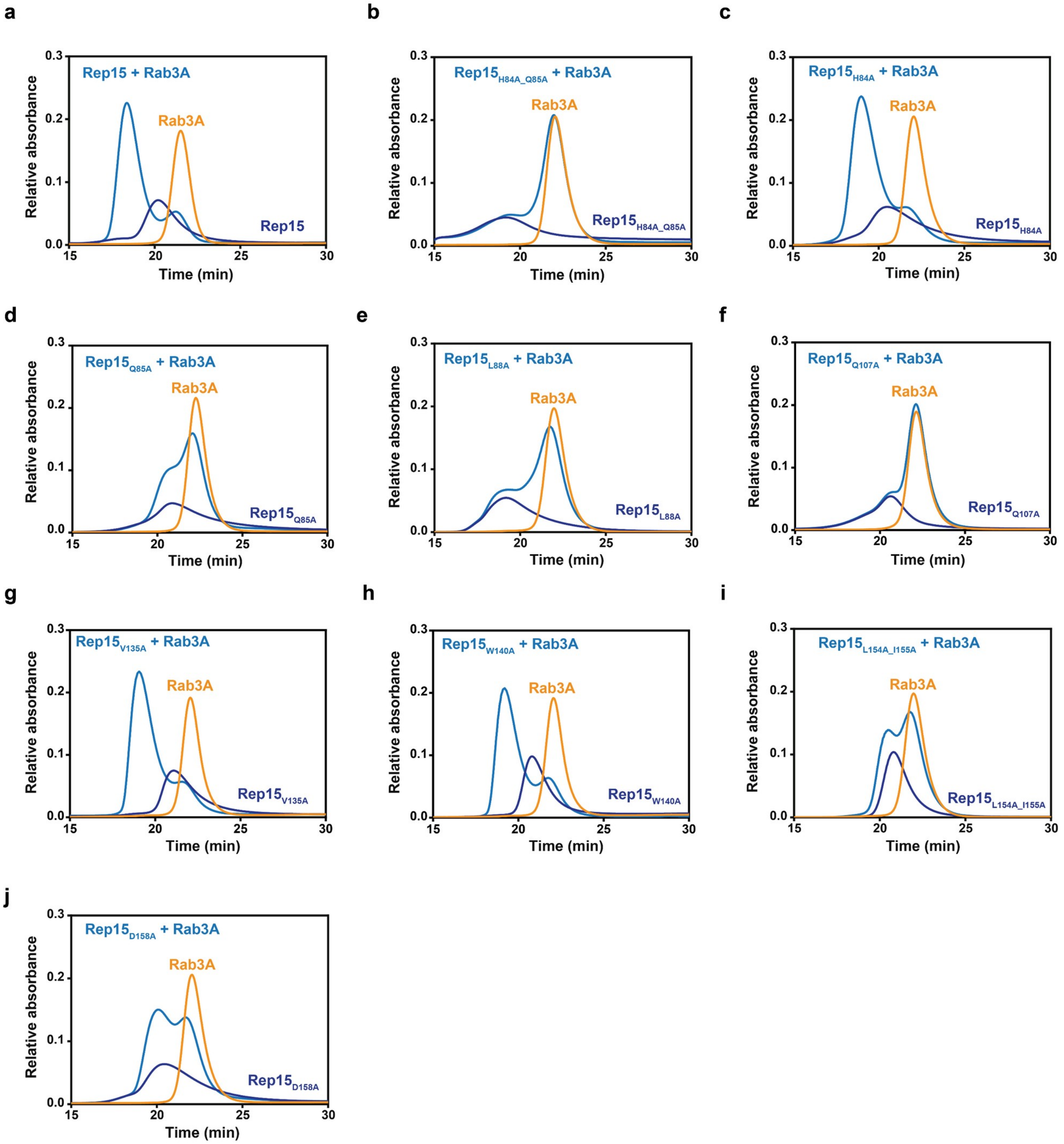

Supplementary Fig. 8: Interaction of Rep15 mutants with Rab3A. a-j The binding of $Rab3A_{GppNHp}$ (orange) with different Rep15 mutants (blue) was systematically tested onto a Superdex75 10/300 GL column. Mutants H84A, V135A and W140A form clear complexes. The Rep15 constructs having low Rab3A binding affinity (H84A_Q85A, Q85A, L88A, Q107A, L154A_I155A and D158A) failed to form stable complexes. The data are representative of at least three repetitions.

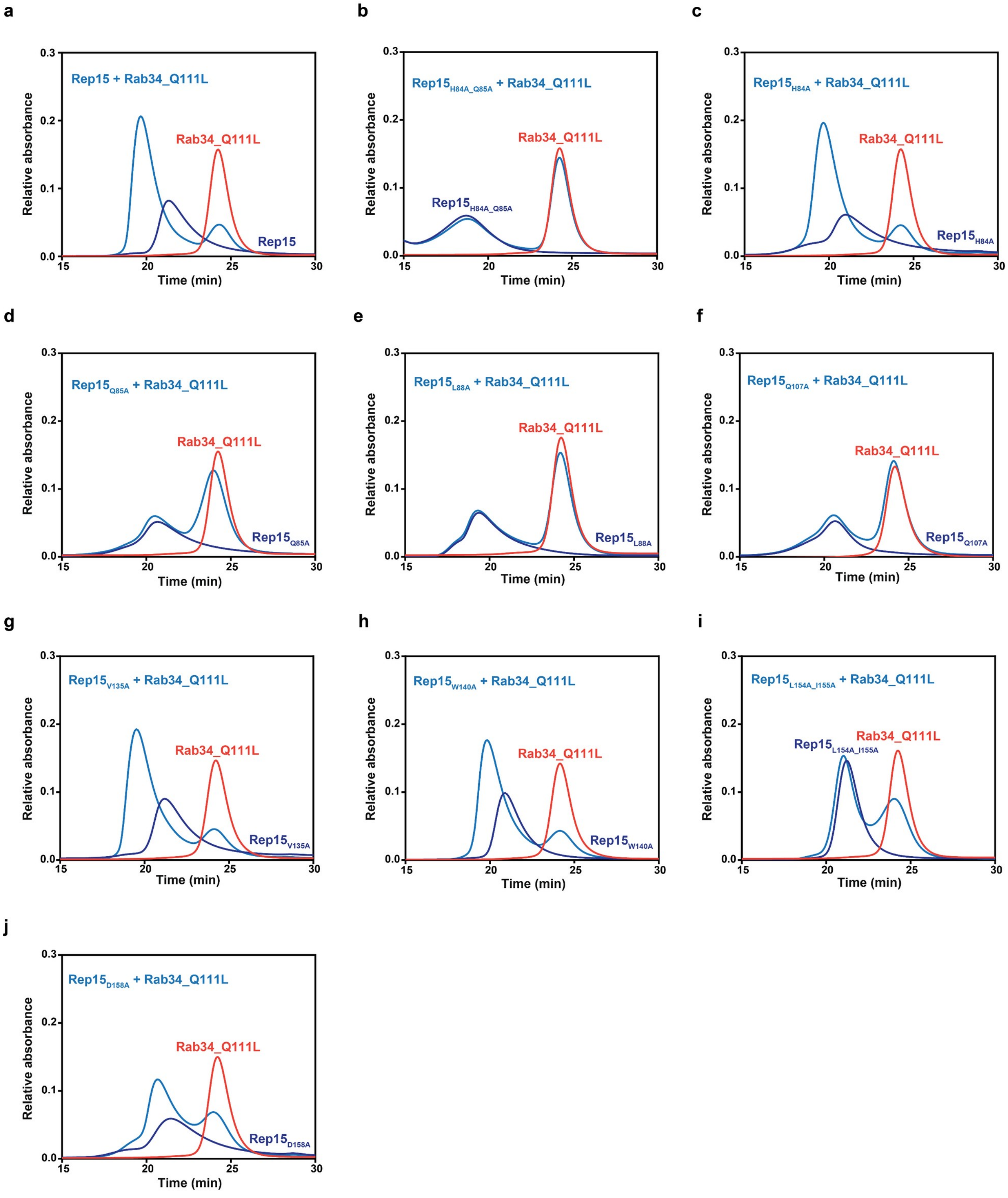

Supplementary Fig. 9: Interaction of Rep15 mutants with Rab34. a-j The binding of Rab34$_{Q111L_1-237_GTP}$ (red) with different Rep15 mutants (blue) was systematically tested onto a Superdex75 10/300 GL column. Mutants H84A, V135A and W140A form clear complexes. The Rep15 constructs H84A_Q85A, Q85A, L88A, Q107A, L154A_I155A and D158A failed to form stable complexes with Rab34$_{Q111L_1-237_GTP}$. The data are representative of at least three repetitions.

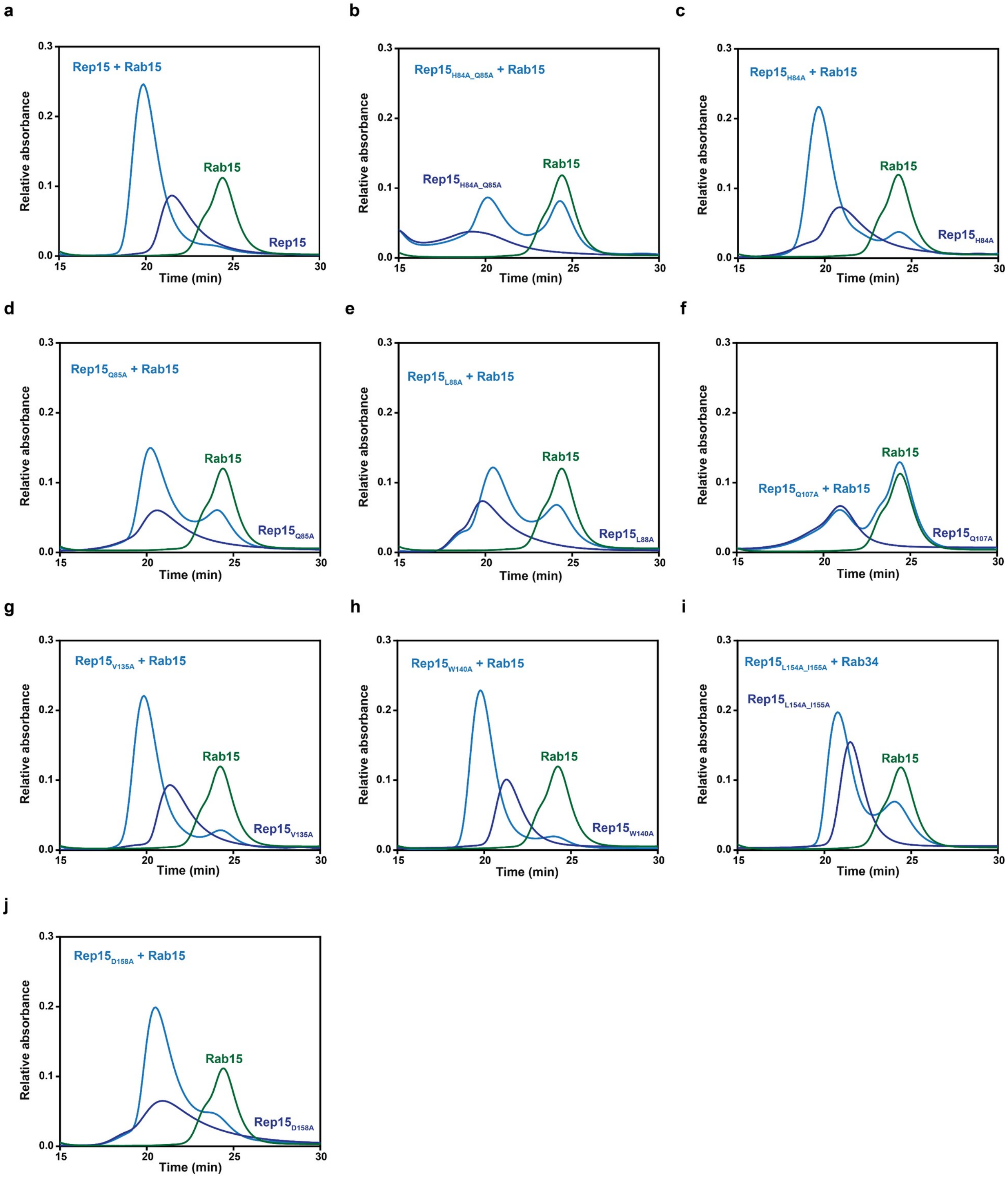

Supplementary Fig. 10: Interaction of Rep15 mutants with Rab15. a-j The binding of Rab15$_{GppNHp}$ (green) with different Rep15 mutants (blue) was systematically tested onto a Superdex75 10/300 GL column. Mutants H84A, V135A, W140A and D158A form clear complexes. The Rep15 constructs H84A_Q85A, Q85A, L88A, Q107A and L154A_I155A failed to form stable complexes with Rab15$_{GppNHp}$. The data are representative of at least three repetitions.

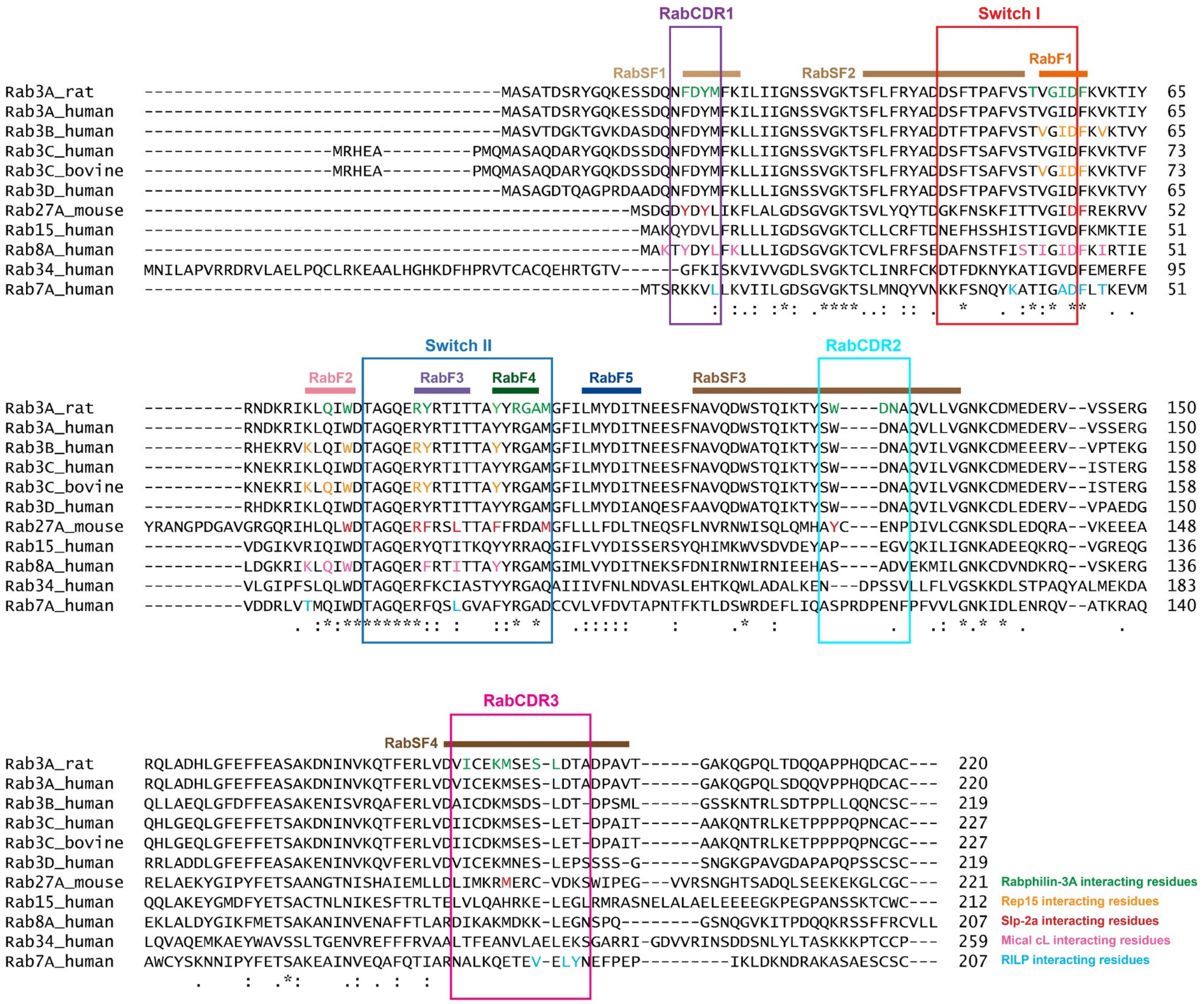

Supplementary Fig. 11: The sequence alignments of Rab3 paralogs with Rab7A, Rab8A, Rab15, Rab27A and Rab34 using Clustal Omega[5]. RabCDRs are shown in purple, cyan and pink boxes. Identical residues are denoted by * and similar residues by :. Switch I and switch II are shown in red and blue boxes. RabSF1-4 motifs are shown in light brown to dark brown. RabF1, RabF2, RabF3, RabF4 and RabF5 motifs are shown in orange, pink, purple, green and blue, respectively. The residues involved in binding with the effector molecules are shown in different colors.

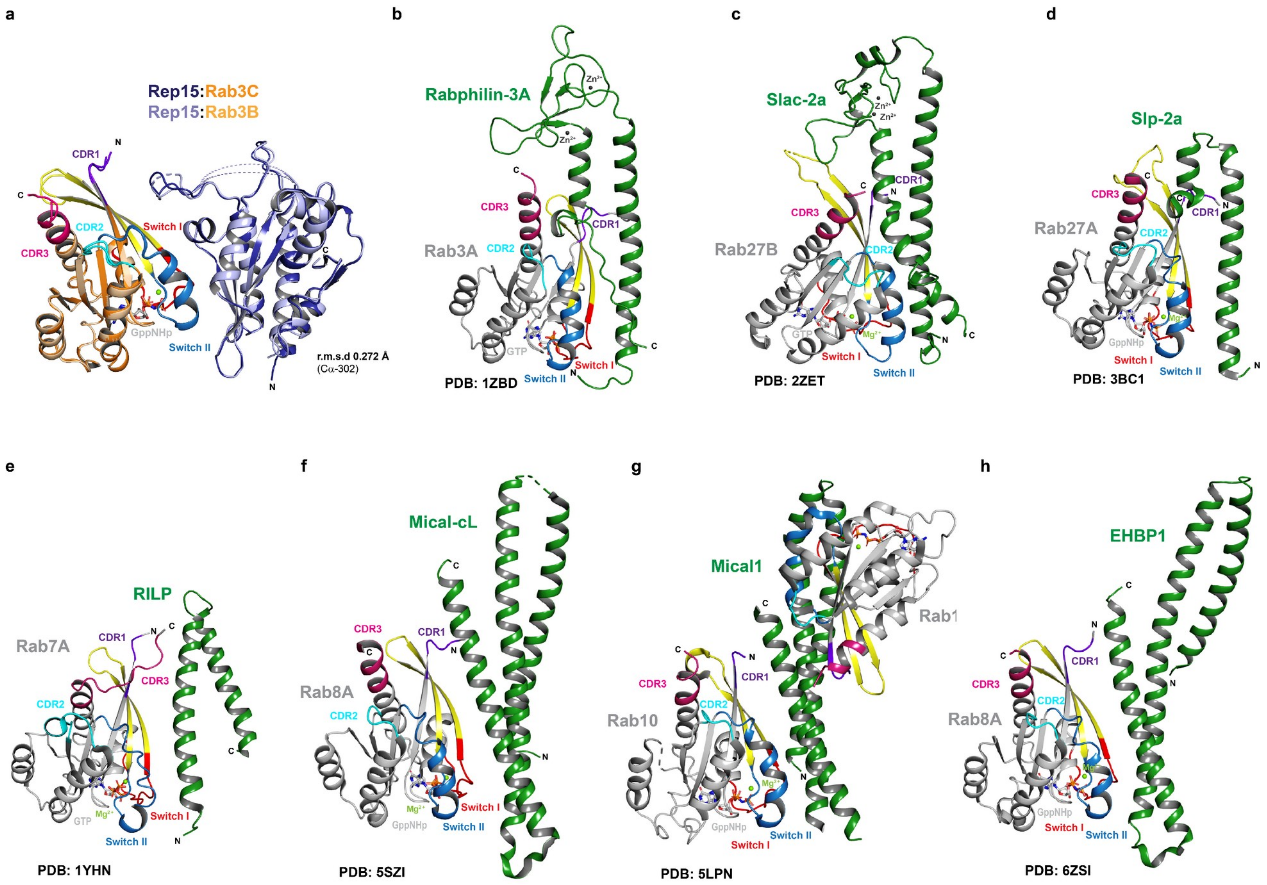

Supplementary Fig. 12: Comparison of Rep15:Rab3B/C complex structure with previously solved Rab3/7/8/27:effector structures. a Superposition of the Rep15:Rab3B$_{GppNHp}$ complex and Rep15:Rab3C $_{GppNHp\_Q222H\_10-227}$ (bovine) complex structures. **b-h** Cartoon representation of the previously solved Rab:effector complex structures. **b** Rabphilin-3A:Rab3A complex[6]. **c** Slac-2a:Rab27B complex[7]. **d** Slp-2a:Rab27A complex[8]. **e** RILP:Rab7A complex[9]. **f** Mical-cL:Rab8A complex[10]. **g** Mical1:Rab10 complex[10]. **h** EHBP1:Rab8A complex[11]. Switch I and switch II regions are shown in red and sky blue, respectively. CDR1, CDR2 and CDR3 regions are shown in purple, cyan and pink color, respectively. Mg^{2+} ion is shown as a green sphere and GppNHp/GTP is shown in stick representation.

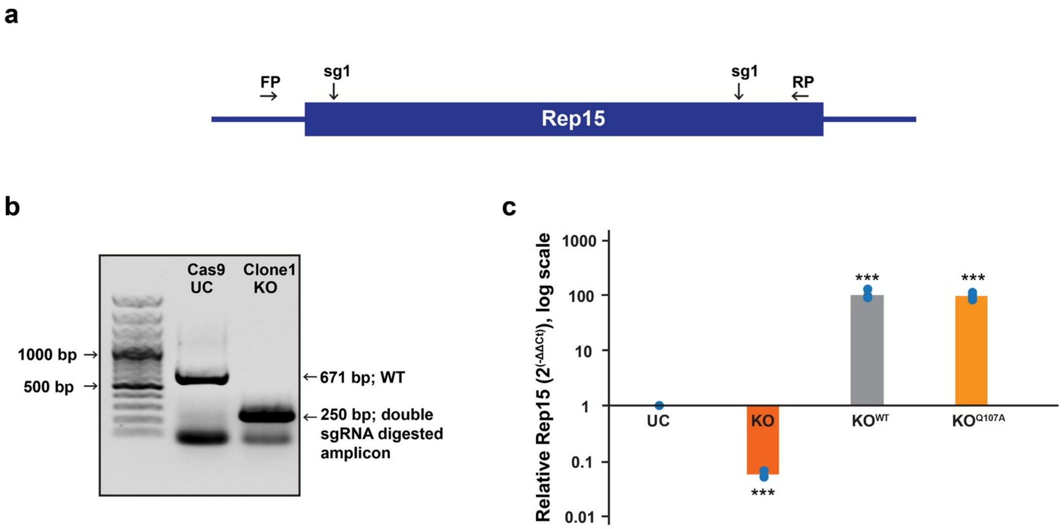

Supplementary Fig. 13: Strategy for the generation of Rep15 KO and validation. **a** Schematic representation of Rep15 genomic locus, the position of the sgRNA and PCR primers used for identifying KO clones. **b** PCR primers were designed to produce 671 bp product in the WT cells, successfully edited homozygous cells produce a smaller product corresponding to ~250 bp. **c** Quantitative Real-time PCR showed a significant reduction in mRNA levels corresponding to Rep15 in the KO cells, with a subsequent increase in the mRNA levels in the two rescue cell lines. Data presented as mean +/- SEM (n = 4; biological repeats). ***p< 0.001, (unpaired, two tailed, Student's t-test). Source data are provided as a Source Data file. The exact p values are provided in the Source data file.

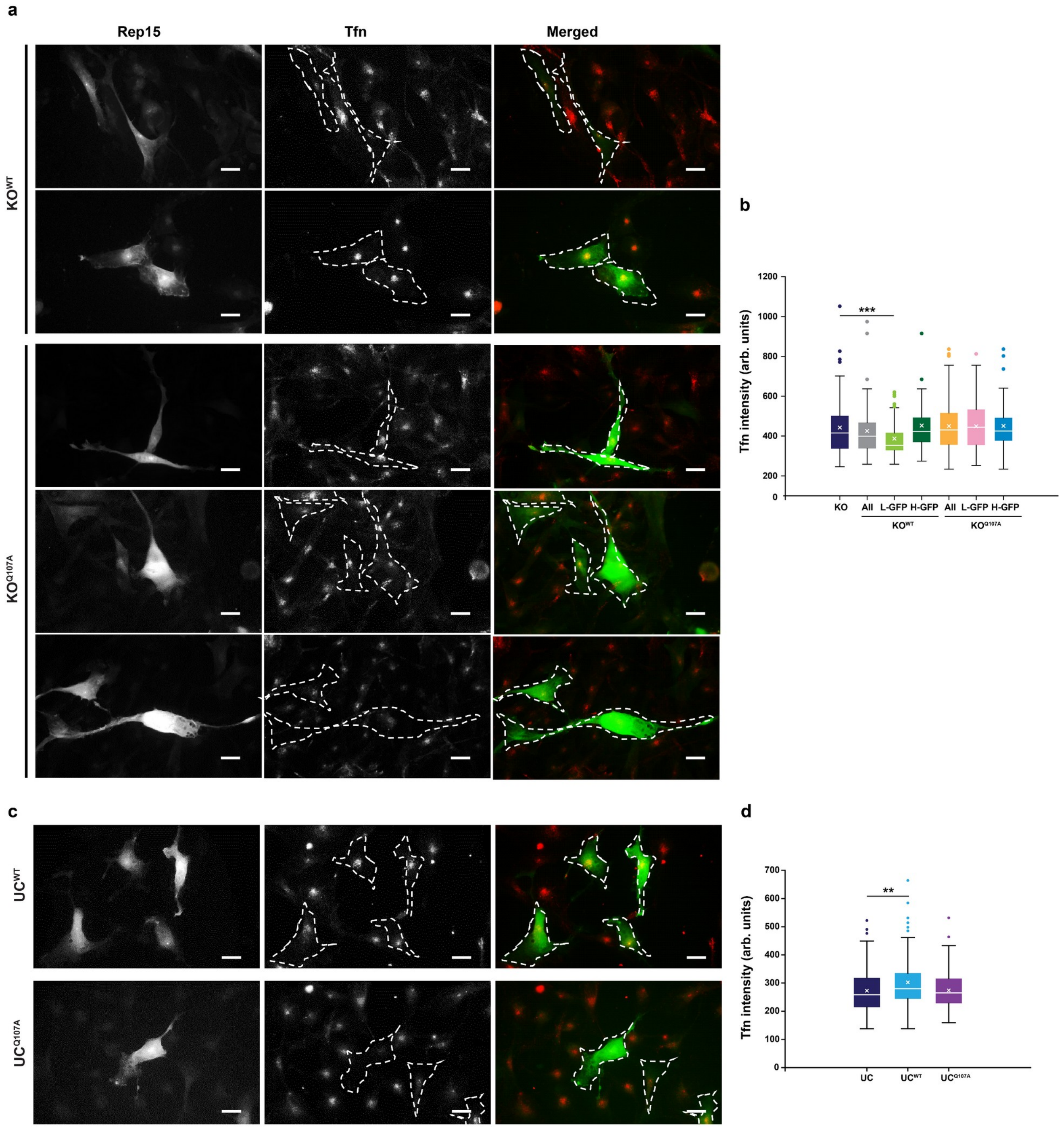

Supplementary Fig. 14: Overexpression of the Rep15 inhibits the recycling pathway. **a** shows representative pictures of the Rep15-KO cells (KO) transfected either with GFP tagged WT Rep-15 (KO^{WT}; upper two-panel) or Q107A mutant (KO^{Q107A}; lower three panels) post 30 min. internalization and 2 h chase. Scale bar, 10 µm. **b** Quantification of transferrin fluorescence shows no significant difference compared to the KO cells when all the cells were considered (All; grey and yellow bars), while when cells were grouped under low and high-GFP expressing cells (corresponding to low and high-Rep15 expression), a significant decrease in the transferrin fluorescence was observed in case of cells transfected with low levels of WT-Rep15 (light green bar). Further, the distribution of the mutant (Q107A) transfected cells into high and low Rep15 expressing cells did not look different

from the control (KO) cells (n=50; cells were examined over three independent experiments). **c** Shows the representative pictures of WT U138MG cells (UC) transfected with either GFP tagged WT Rep-15 (upper panel) or Q107A mutant (lower panel) after 2 h chase post 30 min. internalization. Scale bar, 10 µm. **d** Quantification of transferrin fluorescence shows a significant increase in intensity upon transfection with the WT-Rep15 (UC^{WT}; light blue bar), while Q107A (UC^{Q107A}; purple bar) transfected cells did not look different from the control cells (n=80; cells were examined over three independent experiments). Box plot, shows mean (cross), median (line), 25th and 75th percentile (box). The whiskers extend to the most extreme data points not considered outliers, and the outliers are represented as dots. **p< 0.01, ***p< 0.001, (unpaired, two tailed, Student's t-test). Source data are provided as a Source Data file. n represent the number of cells analysed for the transferrin assay. The exact p values are provided in the Source data file.

Supplementary Table 1: Data-collection and refinement statistics (values in parentheses are for the outer shell).

	**Rep15:Rab3C$_{Q222H_10\text{-}227}$**	**Rep15:Rab3B**	**Rep15 ΔN1:Rab3B$_{Q81L_18\text{-}190}$**
**Data collection#**			
X-Ray Source	X10SA at SLS	X10SA at SLS	X10SA at SLS
Wavelength (Å)	0.977930	0.999961	0.977930
Resolution range (Å)	45.97 - 2.52 (2.61 - 2.52)	47.10 - 2.75 (2.848 - 2.75)	49.54 - 2.8 (2.9 - 2.8)
Space group	I 2 2 2	C 1 2 1	P 1 21 1
Unit cell<br>a,b,c (Å)<br>α, β, γ (°)	58.29, 108.795, 172.027<br>90, 90, 90	188.81, 59.22, 109.02<br>90, 111.905, 90	59.84, 107.96, 91.1<br>90, 106.467, 90
Total reflections	250236 (24166)	198488 (19187)	187605 (19247)
Unique reflections	18879 (1827)	29437 (2888)	27435 (2719)
Multiplicity	13.3 (13.2)	6.7 (6.6)	6.8 (7.1)
Completeness (%)	99.21 (99.19)	99.84 (99.90)	99.68 (99.49)
Mean I/sigma(I)	18.83 (2.05)	14.46 (1.98)	15.80 (1.28)
Wilson B-factor	58.22	67.73	75.21
R_{merge} (%)	0.1095 (1.365)	0.1323 (1.611)	0.09116 (1.412)
R_{meas} (%)	0.1139 (1.42)	0.1435 (1.748)	0.0987 (1.524)
CC1/2	0.999 (0.713)	0.998 (0.615)	0.999 (0.719)
CC*	1 (0.913)	0.999 (0.873)	1 (0.915)
**Refinement**			
Resolution range (Å)	45.97 - 2.52 (2.61 - 2.52)	47.10 - 2.75 (2.848 - 2.75)	49.54 - 2.8 (2.9 - 2.8)
Reflections used in refinement	18793 (1826)	29413 (2885)	27375 (2713)
Reflections used for R-free	941 (90)	1472 (144)	1370 (136)
R_{work} (%)	0.2068 (0.3342)	0.2178 (0.3810)	0.2231 (0.3752)
R_{free} (%)	0.2546 (0.3769)	0.2656 (0.4178)	0.2687 (0.3989)
Number of non-hydrogen atoms<br>Macromolecules<br>Ligands<br>water	3189<br>3116<br>33<br>40	6209<br>6105<br>66<br>38	6145<br>6058<br>66<br>21
R.m.s deviations<br>Bond length (Å)<br>Bond angles (°)	 0.004<br>0.74	 0.006<br>1.09	 0.004<br>0.66
Ramachandran plot<br>Favored (%)<br>Additionally allowed (%)<br>Outliers (%)	 95.44<br>4.56<br>0.00	 94.09<br>5.91<br>0.00	 95.11<br>4.76<br>0.14
B-factors (Å^2)<br>Protein<br>Ligands<br>Water	74.16<br>74.54<br>51.36<br>63.62	77.94<br>78.27<br>51.62<br>69.94	93.92<br>94.25<br>69.08<br>76.97
PDB ID	8A4A	8A4C	8A4B

All data sets were collected from one single crystal on beamline X10SA of the Swiss Light Source (Paul Scherrer Institute, Villigen, Switzerland).

Supplementary Table 2: Expression constructs used in this study.

Construct	Plasmid	Description	Insert boundaries (restriction sites)	Purification	Purpose
His-MBP-Rep15	pMAL-hs-Rep15	6xHis-MBP-TEV-hs-Rep15 (fl)	aa 1-236 (fl) (NdeI / XhoI)	Ni-NTA, TEV, Ni-NTA, Superdex 75	aSEC, ITC, stopped-flow, crystallization
His-Rep15	pET19mod-hs-Rep15	6xHis-TEV-hs-Rep15 (fl)	aa 1-236 (fl) (NdeI / XhoI)	Ni-NTA, TEV, Ni-NTA, Superdex 75	Co-purification with Rab, crystallization
His- Rep15 ΔN1	pET19mod-hs-Rep15 ΔN1	6xHis-TEV-hs-Rep15 ΔN1	aa 17-236 (NdeI / XhoI)	Ni-NTA, TEV, Ni-NTA, Superdex 75	aSEC, crystallization
His-MBP-Rep15 ΔN1	pMAL-hs-Rep15 ΔN1	6xHis-MBP-TEV-hs-Rep15 ΔN1	aa 17-236 (NdeI / XhoI)	Ni-NTA, TEV, Ni-NTA, Superdex 75	aSEC, crystallization
His-MBP-Rep15 ΔNC1	pMAL-hs-Rep15 ΔNC1	6xHis-MBP-TEV-hs-Rep15 ΔNC1	aa 17-115 (NdeI / XhoI)	Ni-NTA, TEV	purification
His-MBP-Rep15 ΔN2	pMAL-hs-Rep15 ΔN2	6xHis-MBP-TEV-hs-Rep15 ΔN2	aa 140-236 (NdeI / XhoI)	Ni-NTA, TEV	purification
His-MBP-Rep15 ΔNC2	pMAL-hs-Rep15 ΔNC2	6xHis-MBP-TEV-hs-Rep15 ΔNC2	aa 17-202 (NdeI / XhoI)	Ni-NTA, TEV	purification
His-Rab15	pET19mod-hs-Rab15$_{opti}$	Rai *et al*., 2016[10]	aa 1-212 (fl) Codon optimized (NdeI / XhoI)	Ni-NTA, TEV, Ni-NTA, Superdex75	aSEC, ITC, crystallization
Rab15$_{Q67L_1-176}$	pET30-hs-Rab15$_{Q67L_1-176_opti}$	Rab15$_{Q67__1-176_opti}$ (tag free)	aa 1-176 Codon optimized (NdeI / XhoI) Quick change to mutate Q67 to L	Ni-NTA, TEV, Ni-NTA, Superdex75	Co-purification with Rep15-ΔN1, crystallization
GAL4_AD-HA-Rep15	pGADT7-hs-Rep15	GAL4_AD-HA-hs-Rep15	aa 1-236 (fl) (NdeI / XhoI)		Yeast-two hybrid
GAL4_AD-HA	pGADT7	Clonetech			Yeast-two hybrid
Rab$_{QL}$ (DA) library	pLex hs Rab QL	Lindsay *et al*., 2013[12]	Rab library Q to L (dominant active)		Yeast-two hybrid
LexA_BD	pLexA	Clontech			Yeast-two hybrid
LexA_BD-Rab3A	pLexA hs Rab3A	Lindsay *et al*., 2013[12]	aa 1-220 (fl)		Yeast-two hybrid
LexA_BD-Rab3A$_{Q81L}$	pLexA hs Rab3A$_{Q81L}$	Lindsay *et al*., 2013[12]	aa 1-220 (fl) Q81 to L		Yeast-two hybrid
LexA_BD-Rab3A$_{T36N}$	pLexA hs Rab3A$_{T36N}$	Lindsay *et al*., 2013[12]	aa 1-220 (fl) T36 to N		Yeast-two hybrid
LexA_BD-Rab3B	pLexA hs Rab3B	Lindsay *et al*., 2013[12]	aa 1-219 (fl)		Yeast-two hybrid
LexA_BD-Rab3B$_{Q81L}$	pLexA hs Rab3B$_{Q81L}$	Lindsay *et al*., 2013[12]	aa 1-219 (fl) Q81 to L		Yeast-two hybrid
LexA_BD-Rab3B$_{T36N}$	pLexA hs Rab3B$_{T36N}$	Lindsay *et al*., 2013[12]	aa 1-219 (fl) T36 to N		Yeast-two hybrid
LexA_BD-Rab3C	pLexA hs Rab3C	Lindsay *et al*., 2013[12]	aa 1-227 (fl)		Yeast-two hybrid
LexA_BD-Rab3C$_{Q89L}$	pLexA hs Rab3C$_{Q89L}$	Lindsay *et al*., 2013[12]	aa 1-227 (fl) Q89 to L		Yeast-two hybrid
LexA_BD-Rab3C$_{T44N}$	pLexA hs Rab3C$_{T44N}$	Lindsay *et al*., 2013[12]	aa 1-227 (fl) T44 to N		Yeast-two hybrid
LexA_BD-Rab3D	pLexA hs Rab3D	Lindsay *et al*., 2013[12]	aa 1-219 (fl)		Yeast-two hybrid

LexA_BD-Rab3D$_{Q81L}$	pLexA hs Rab3D$_{Q81L}$	Lindsay *et al.*, 2013[12]	aa 1-219 (fl) Q81 to L		Yeast-two hybrid
LexA_BD-Rab3D$_{T36N}$	pLexA hs Rab3D$_{T36N}$	Lindsay *et al.*, 2013[12]	aa 1-219 (fl) T36 to N		Yeast-two hybrid
LexA_BD-Rab34	pLexA hs Rab34	Lindsay *et al.*, 2013[12]	aa 1-259 (fl)		Yeast-two hybrid
LexA_BD-Rab34$_{Q111L}$	pLexA hs Rab34$_{Q81L}$	Lindsay *et al.*, 2013[12]	aa 1-259 (fl) Q111 to L		Yeast-two hybrid
LexA_BD-Rab34$_{T66N}$	pLexA hs Rab34$_{T66N}$	Lindsay *et al.*, 2013[12]	aa 1-259 (fl) T66 to N		Yeast-two hybrid
EGFP-Rep15	pEGFP (C1) Rep15	EGFP-hs-Rep15	aa 1-236 (fl) (XhoI/SalI)		Transient expression
mCherry-Rab3A$_{Q81L}$	pmCherry (C1) Rab3A$_{Q81L}$	mCherry-hs-Rab3A$_{Q81L}$	aa 1-220 (fl) (XhoI/BamH1)		Transient expression
mCherry-Rab3B$_{Q81L}$	pmCherry (C1) Rab3B$_{Q81L}$	mCherry-hs-Rab3B$_{Q81L}$	aa 1-219 (fl) (XhoI/BamH1)		Transient expression
mCherry-Rab3C$_{Q89L}$	pmCherry (C1) Rab3C$_{Q89L}$	mCherry-hs-Rab3C$_{Q89L}$	aa 1-227 (fl) (XhoI/BamH1)		Transient expression
mCherry-Rab3D$_{Q81L}$	pmCherry (C1) Rab3D$_{Q81L}$	mCherry-hs-Rab3D$_{Q81L}$	aa 1-219 (fl) (XhoI/BamH1)		Transient expression
His-Rab3A	pET19mod-hs-Rab3A	6xHis-TEV-hs-Rab3A	aa 1-220 (fl) (NdeI / XhoI)	Ni-NTA, TEV, Ni-NTA, Superdex 75	aSEC, ITC, stopped-flow, crystallization
His-Rab3A$_{2-220}$	pOPINE-mm-Rab3A$_{2-220}$	6xHis-mm-Rab3A$_{2-220}$	aa 2-220	Ni-NTA, Superdex 75	aSEC, crystallization
His-Rab3B	pET19mod-hs-Rab3B	6xHis-TEV-hs-Rab3B	aa 1-219 (fl) (NdeI / XhoI)	Ni-NTA, TEV, Ni-NTA, Superdex 75	aSEC, ITC, crystallization
His-Rab3A$_{V61N}$	pET19mod-hs-Rab3A$_{V61N}$	6xHis-TEV-hs-Rab3A$_{V61N}$	aa 1-220 (fl) (NdeI / XhoI) Quick change to mutate V61 to N	Ni-NTA, TEV, Ni-NTA, Superdex 75	aSEC, ITC
His-Rab3A$_{Y84F}$	pET19mod-hs-Rab3A$_{Y84F}$	6xHis-TEV-hs-Rab3A$_{Y84F}$	aa 1-220 (fl) (NdeI / XhoI) Quick change to mutate Y84 to F	Ni-NTA, TEV, Ni-NTA, Superdex 75	aSEC, ITC
His-Rab3C$_{Q222H_10-227}$	pOPINE-bt-Rab3C$_{Q222H__10-227}$	6xHis-bt-Rab3C$_{Q222H__10-227}$	aa 10-227 mutation Q222H	Ni-NTA, Superdex 75	aSEC, crystallization
His-Rab3C	pET19mod-hs-Rab3C	6xHis-TEV-hs-Rab3C	aa 1-227 (fl) (NdeI / XhoI)	Ni-NTA, TEV, Ni-NTA, Superdex 75	aSEC, ITC, crystallization
His-Rab3D	pET19mod-hs-Rab3D	6xHis-TEV-hs-Rab3D	aa 1-219 (fl) (NdeI / XhoI)	Ni-NTA, TEV, Ni-NTA, Superdex 75	aSEC, ITC, crystallization
His-MBP-Rab34$_{Q111L_1-237}$	pMAL-hs-Rab34$_{Q111L_1-237}$	6xHis-MBP-TEV-hs-Rab34$_{Q111L_1-237}$	aa 1-237 (NdeI / XhoI)	Ni-NTA, TEV, Ni-NTA, Superdex 75	aSEC, ITC
His-MBP-Rab34$_{Q111L_F114Y_1-237}$	pMAL-hs-Rab34$_{Q111L_F114Y_1-237}$	6xHis-MBP-TEV-hs-Rab34$_{Q111L_F114Y_1-237_1-237}$	aa 1-237 (NdeI / XhoI)	Ni-NTA, TEV, Ni-NTA, Superdex 75	aSEC, ITC
Rab3A$_{Q81L_19-217}$	pET30a-hs-Rab3A$_{Q81L_19-217}$	hs- Rab3A$_{Q81L_19-217}$ (tag free)	aa 19-217 (NdeI / XhoI) Quick change to mutate Q81 to L	Ni-NTA, TEV, Ni-NTA, Superdex 75	Co-purification with Rep15 ΔN1, crystallization
Rab3B$_{Q81L_18-190}$	pET30a-hs-Rab3B$_{Q81L_18-190}$	hs- Rab3B$_{Q81L_18-190}$ (tag free)	aa 18-190 (NdeI / XhoI) Quick change to mutate Q81 to L	Ni-NTA, TEV, Ni-NTA, Superdex 75	Co-purification with Rep15 ΔN1, crystallization
Rab34$_{Q111L_1-237}$	pET30a-hs-Rab34$_{Q111L_1-237}$	hs- Rab34$_{Q111L_1-237}$ (tag free)	aa 1-237 (NdeI / XhoI) Quick change to	Ni-NTA, TEV, Ni-NTA, Superdex 75	Co-purification with Rep15

			mutate Q111 to L		ΔN1, crystallization
His-MBP-Rep15$_{H84A_Q85A}$	pMAL-hs-Rep15$_{H84A_Q85A}$	6xHis-MBP-TEV-hs-Rep15$_{H84A_Q85A}$	aa 1-236 (fl) (NdeI / XhoI) Quick change to mutate H84 to A and Q85 to A	Ni-NTA, TEV, Ni-NTA, Superdex 75	aSEC, ITC
His-MBP-Rep15$_{H84A}$	pMAL-hs-Rep15$_{H84A}$	6xHis-MBP-TEV-hs-Rep15$_{H84A}$	aa 1-236 (fl) (NdeI / XhoI) Quick change to mutate H84 to A	Ni-NTA, TEV, Ni-NTA, Superdex 75	aSEC, ITC
His-MBP-Rep15$_{Q85A}$	pMAL-hs-Rep15$_{Q85A}$	6xHis-MBP-TEV-hs-Rep15$_{Q85A}$	aa 1-2236 (fl) (NdeI / XhoI) Quick change to mutate Q85 to A	Ni-NTA, TEV, Ni-NTA, Superdex 75	aSEC, ITC
His-MBP-Rep15$_{L88A}$	pMAL-hs-Rep15$_{L88A}$	6xHis-MBP-TEV-hs-Rep15$_{L88A}$	aa 1-236 (fl) (NdeI / XhoI) Quick change to mutate L88 to A	Ni-NTA, TEV, Ni-NTA, Superdex 75	aSEC, ITC
His-MBP-Rep15$_{Q107A}$	pMAL-hs-Rep15$_{Q107A}$	6xHis-MBP-TEV-hs-Rep15$_{Q107A}$	aa 1-236 (fl) (NdeI / XhoI) Quick change to mutate Q107 to A	Ni-NTA, TEV, Ni-NTA, Superdex 75	aSEC, ITC
His-MBP-Rep15$_{V135A}$	pMAL-hs-Rep15$_{V135A}$	6xHis-MBP-TEV-hs-Rep15$_{V135A}$	aa 1-236 (fl) (NdeI / XhoI) Quick change to mutate V135 to A	Ni-NTA, TEV, Ni-NTA, Superdex 75	aSEC, ITC
His-MBP-Rep15$_{W140A}$	pMAL-hs-Rep15$_{W140A}$	6xHis-MBP-TEV-hs-Rep15$_{W140A}$	aa 1-236 (fl) (NdeI / XhoI) Quick change to mutate W140 to A	Ni-NTA, TEV, Ni-NTA, Superdex 75	aSEC, ITC
His-MBP-Rep15$_{L154A_I155A}$	pMAL-hs-Rep15$_{L154A_I155A}$	6xHis-MBP-TEV-hs-Rep15 $_{L154A\_I155A}$	aa 1-236 (fl) (NdeI / XhoI) Quick change to mutate L154 to A and I155 to A	Ni-NTA, TEV, Ni-NTA, Superdex 75	aSEC, ITC
His-MBP-Rep15$_{D158A}$	pMAL-hs-Rep15$_{D158A}$	6xHis-MBP-TEV-hs-Rep15$_{D158A}$	aa 1-236 (fl) (NdeI / XhoI) Quick change to mutate D158 to A	Ni-NTA, TEV, Ni-NTA, Superdex 75	aSEC, ITC
Rab8A$_{1-176}$	pET19mod-hs-Rab8A$_{1-176_opti}$	Rai *et al.*, 2016[10]	aa 1-176 Codon optimized (NdeI / XhoI)	Ni-NTA, TEV, Ni-NTA, Superdex75	aSEC
His-MBP-Rab27A	pMAL-hs-Rab27A	6xHis-MBP-TEV-hs-Rab27A	aa 1-221 (fl) Codon optimized (NdeI/XhoI)	Ni-NTA, TEV, Ni-NTA, Superdex75	aSEC
His-MBP-Rab27A$_{F81Y}$	pMAL-hs-Rab27A$_{F81Y}$	6xHis-MBP-TEV-hs-Rab27A$_{F81Y}$	aa 1-221 (fl) Codon optimized (NdeI/XhoI) Quick change to mutate F81 to Y	Ni-NTA, TEV, Ni-NTA, Superdex75	aSEC

aa: amino acid, hs: homo sapiens, mm: mus musculus, bt: bos taurus, MBP: maltose binding protein, fl: full-length

Supplementary Table 3: Primers used in this study.

Primer name	Primer sequence (5´ to 3´)
REP15_Nde1_f	GATCCATATGGGGCAGAAAGCATCGCAACAGTTGGCTCTGAAGGAC
REP15_Xho1_r	GATCCTCGAGTCAGAGAATGCTGATATAAACCTTGCCCACCTCTCT
Rep15_dN1__NdeI_f	GATCCATATGGTGCCCGTCGTCTGTGAGGTGGTCAGTGAAGCT
Rep15_dC1_XhoI_r	GATCCTCGAGTCACATCTGCAGAGTCTGTACTGCGAGCTGAAG
Rep15_dN2__NdeI_f	GATCCATATGTGGAAGAAAAGTAGATTTGATAAGCTGGAA
REP15_dC2_Xho1_r	GATCCTCGAGTCAGACAAACTGAGCCACAGCCTTCCCTCCTGG
Rab15_Nde1_f	AAAAACATATGGCCAAACAGTATGACGTTCTGTTCCG
Rab15_XhoI_r	AAAAAACTCGAGTCATTCTTTACGATGGGCTTGCAGAACCAGC
Rep15_XhoI_f	GATCCTCGAGATGGGGCAGAAAGCATCGCAACAGTTGGCTCTG
Rep15_SalI_r	GATCGTCGACTGGAGAATGCTGATATAAACCTTGCCCACCTCTCT
Rab3A_Xho1_f	GATCCTCGAGCTATGGCATCCGCCACAGACTCGCGCTATG
Rab3A_BamH1_r	GATCGGATCCTCAGCAGGCGCAGTCCTGGTGCGGTGGCAC
Rab3B_Xho1_f	GATCCTCGAGCTATGGCTTCAGTGACAGATGGTAAAACTGGA
Rab3B_BamH1_r	GATCGGATCCCTAGCATGAGCAGTTCTGCTGCAGCAGCGG
Rab3C_Xho1_f	GATCCTCGAGCTATGAGACACGAAGCGCCCATGCAGATGGCC
Rab3C_BamH1_r	GATCGGATCCCTAGCAGGCACAGTTGGGCTGCGGTGGAGG
Rab3D_Xho1_f	GATCCTCGAGCTATGGCATCAGCTGGAGACACCCAGGCAGGC
Rab3D_BamH1_r	GATCGGATCCCTAGCAGCTGCAGCTGCTGGGCTGGGGGGC
Rab34_Xho1_f	GATCCTCGAGCTATGAACATTCTGGCACCCGTGCGGAGGGATCGC
Rab34_BamH1_r	GATCGGATCCTCAGCAACATGTGGGCTTCTTCTTGCTGGC
Rab3A_Nde1_f	GATCCATATGGCATCCGCCACAGACTCGCGCTATG
Rab3A_Xho1_r	GATCCTCGAGTCAGCAGGCGCAGTCCTGGTGCGGTGGCAC
Rab3A_dNC_Nde1_f	GATCCATATGTTCGACTACATGTTCAAGATTCTCATCATC
Rab3A_dNC_Xho1_r	GATCCTCGAGTCAGTCCTGGTGCGGTGGCACCTGCTGGTCACT
Rab3B_Nde1_f	GATCCATATGGCTTCAGTGACAGATGGTAAAACTGGA
Rab3B_Xho1_r	GATCCTCGAGCTAGCATGAGCAGTTCTGCTGCAGCAGCGG
Rab3B_dNC_Nde1_f	GATCCATATGAATTTTGACTACATGTTTAAACTGCTTATC
Rab3B_dNC_Xho1_r	GATCCTCGAGCTACGAATCAGACATCTTGTCACAAATGGCATC
Rab3C_NdeI_f	GATCCATATGAGACACGAAGCGCCCATGCAGATGGCC
Rab3C_Xho1_r	GATCCTCGAGCTAGCAGGCACAGTTGGGCTGCGGTGGAGG
Rab3D_NdeI_f	GATCCATATGGCATCAGCTGGAGACACCCAGGCAGGC
Rab3D_Xho1_r	GATCCTCGAGCTAGCAGCTGCAGCTGCTGGGCTGGGGGGC
Rab34_Nde1_f	GATCCATATGAACATTCTGGCACCCGTGCGGAGGGATCGC
Rab34_dC_Xho_r	AAAAAACTCGAGTCAGACAACATCCCCAATGCGTCGAG
Rab34_Q111L_F114Y_f	ACCGCTGGGCTAGAGAGGTACAAATGCATTGCATCAACC
Rab34_Q111L_F114Y_r	GGTTGATGCAATGCATTTGTACCTCTCTAGCCCAGCGGT
Rep15_H84A_Q85A_f	ACCACCACCAAGATGACCAAGGCCGCAGCCTTCCTGTTTGGTGCAGACTGG
Rep15_H84A_Q85A_r	CCAGTCTGCACCAAACAGGAAGGCTGCGGCCTTGGTCATCTTGGTGGTGGT
Rep15_H84A_f	ACCACCACCAAGATGACCAAGGCCCAAGCCTTCCTGTTTGGTGC
Rep15_H84A_r	GCACCAAACAGGAAGGCTTGGGCCTTGGTCATCTTGGTGGTGGT
Rep15_Q85A_f	ACCACCAAGATGACCAAGCACGCAGCCTTCCTGTTTGGTGCAGA
Rep15_Q85A_r	TCTGCACCAAACAGGAAGGCTGCGTGCTTGGTCATCTTGGTGGT
Rep15_L88A_f	ATGACCAAGCACCAAGCCTTCGCGTTTGGTGCAGACTGGATTTGG
Rep15_L88A_r	CCAAATCCAGTCTGCACCAAACGCGAAGGCTTGGTGCTTGGTCAT
Rep15_Q107A_f	TCTGACAAGCAAATAAAGCTTGCGCTCGCAGTACAGACTCTGCAG
Rep15_Q107A_r	CTGCAGAGTCTGTACTGCGAGCGCAAGCTTTATTTGCTTGTCAGA
Rep15_V135A_f	TCCAATCCAGAATCAAGGGCAGAGGAGTCTTCCTGGAAG
Rep15_V135A_r	CTTCCAGGAAGACTCCTCTGCCCTTGATTCTGGATTGGA
Rep15_W140A_f	AGGGTAGAGGAGTCTTCCGCGAAGAAAAGTAGATTTGAT
Rep15_W140A_r	ATCAAATCTACTTTTCTTCGCGGAAGACTCCTCTACCCT
Rep15_L154A_I155A_f	AAGCTGGAAGAATTCTGTAACGCAGCAGGAGAGGATTGCCTGGGTCTG
Rep15_L154A_I155A _r	CAGACCCAGGCAATCCTCTCCTGCTGCGTTACAGAATTCTTCCAGCTT
Rep15_D158A_f	TTCTGTAACTTAATAGGAGAGGCTTGCCTGGGTCTGTTTATCATC
Rep15_D158A_r	GATGATAAACAGACCCAGGCAAGCCTCTCCTATTAAGTTACAGAA

Rab3A_V61N_f	ACCGTGGGCATCGACTTCAAGAACAAGACCATCTATCGCAACGAC
Rab3A_V61N_r	GTCGTTGCGATAGATGGTCTTGTTCTTGAAGTCGATGCCCACGGT
Rab3A_Y84F_f	GACACAGCAGGGCAAGAGCGGTTCCGGACCATCACCACCGCATAC
Rab3A_Y84F_r	GTATGCGGTGGTGATGGTCCGGAACCGCTCTTGCCCTGCTGTGTC
Rab27A_F81Y_f	GATACCGCTGGTCAAGAGCGCTAT CGTAGCCTGACAACCGCTTTT
Rab27A_F81Y_r	AAAAGCGGTTGTCAGGCTACGATAGCGCTCTTGACCAGCGGTATC
sg1-Rep15	ACTGACCACCTCACAGACGA
sg2-Rep15	TTAATAGGAGAGGATTGCCT
Rep15_KO_identification_f	GAACCATTAACAGATGTGGCCT
Rep15_KO_identification_r	TGGAGGATATTCAAATCCAAGG
RT-PCR_Rep15_f	TTTGGGGATCCGACAAGCAA
RT-PCR_Rep15_r	ACCCAGGCAATCCTCTCCTA
RT-PCR_Rab3A_f	CAGATCTGGGACACAGCAGG
RT-PCR_Rab3A_r	CTTGTTTCCTACCAGCAGCACCT
RT-PCR_Rab3B_f	GCTGTCCAAGACTGGGCTAC
RT-PCR_Rab3B_r	AAGGCCTGCCTTACACTGATGTT
RT-PCR_Rab3C_f	AGATTTGGGACACAGCAGGC
RT-PCR_Rab3C_r	TGTCACACTTGTTCCCAACCAG
RT-PCR_Rab3D_f	ATCAGGAATCCTTTGCCGCT
RT-PCR_Rab3D_r	TCGAAACCAAGGTCGTCGG
RT-PCR_Rab34_f	GCTCAGTATGCGCTGATGGA
RT-PCR_Rab34_r	TTGATGCGGACAACATCCCC
RT-PCR_Rab15_f	CCAGTCATGGCGAAGCAGTA
RT-PCR_Rab15_r	TATCCGCACTTTGATGCCGTCT
RT-PCR_Alas1_f	CTGCAAAGATCTGACCCCTC
RT-PCR_Alas1_r	CCTCATCCACGAAGGTGATT
RT-PCR_GAPDH_f	ACCCAGAAGACTGTGGATGG
RT-PCR_ GAPDH _r	TTCTAGACGGCAGGTCAGGT

Supplementary Table 4: Thermodynamic parameters of binding experiments measured by ITC

	N (sites)	K_D (M)	ΔG (kcal/mol)	ΔH (kcal/mol)	-TΔS (kcal/mol)
Rab15:Rep15<br>500 µM → 50 µM	0.86 ± 0.01	$0.47 \pm 0.12 \times 10^{-6}$	-8.66	-3.44	-5.22
Rab3A:Rep15<br>500 µM → 50 µM	0.79 ± 0.01	$0.48 \pm 0.04 \times 10^{-6}$	-8.61	-7.16	-1.45
Rab3B:Rep15<br>500 µM → 50 µM	0.92 ± 0.01	$2.16 \pm 0.13 \times 10^{-6}$	-7.73	-5.35	-2.38
Rab3C:Rep15<br>500 µM → 50 µM	0.95 ± 0.01	$0.94 \pm 0.11 \times 10^{-6}$	-8.22	-6.58	-1.64
Rab3D:Rep15<br>500 µM → 50 µM	0.75 ± 0.01	$0.79 \pm 0.08 \times 10^{-6}$	-8.33	-5.39	-2.94
$Rab34_{Q111L\_1\text{-}237}$:Rep15<br>500 µM → 50 µM	1.01 ± 0.1	$64 \pm 32.4 \times 10^{-9}$	-9.81	1.40	-11.21
Rab3A:$Rep15_{H84A\_Q85A}$<br>500 µM → 50 µM	N.A.	N.A.	N.A.	N.A.	N.A.
Rab3A:$Rep15_{H84A}$<br>500 µM → 50 µM	0.92 ± 0.01	$0.45 \pm 0.01 \times 10^{-6}$	-8.66	-5.00	-3.66
Rab3A:$Rep15_{Q85A}$<br>500 µM → 50 µM	0.80 ± 0.07	$18.5 \pm 4.5 \times 10^{-6}$	-6.47	-5.06	-1.41
Rab3A:$Rep15_{L88A}$<br>500 µM → 50 µM	0.95 ± 0.01	$9.7 \pm 0.5 \times 10^{-6}$	-6.84	-3.33	-3.51
Rab3A:$Rep15_{Q107A}$<br>500 µM → 50 µM	N.A.	N.A.	N.A.	N.A.	N.A.
Rab3A:$Rep15_{V135A}$<br>500µM → 50µM	1.05 ± 0.01	$0.51 \pm 0.06 \times 10^{-6}$	-8.57	-5.96	-2.61
Rab3A:$Rep15_{W140A}$<br>500 µM → 50 µM	0.91 ± 0.01	$0.40 \pm 0.01 \times 10^{-6}$	-8.71	-4.78	-3.93
Rab3A:$Rep15_{L154A\_I155A}$<br>500 µM → 50 µM	N.A.	N.A.	N.A.	N.A.	N.A.
Rab3A:$Rep15_{D158A}$<br>500 µM → 50 µM	1.09 ± 0.01	$5.59 \pm 0.2 \times 10^{-6}$	-7.17	-3.09	-4.08
Rab27A:Rep15<br>600 µM → 60 µM	0.89 ± 0.15	$86.2 \pm 18.1 \times 10^{-6}$	-5.53	5.67	-11.2
$Rab27A_{F81Y}$:Rep15<br>600 µM → 60 µM	0.92 ± 0.02	$10.8 \pm 1.8 \times 10^{-6}$	-6.78	2.31	-9.09
$Rab34_{Q111L\_F114Y\_1\text{-}237}$:Rep15<br>600 µM → 60 µM	1.05 ± 0.15	$78.7 \pm 9.5 \times 10^{-9}$	-9.69	-4.03	-5.66
$Rab3A_{V61N}$:Rep15	0.75 ± 0.01	$0.39 \pm 0.6 \times 10^{-6}$	-8.73	-6.25	-2.48

500 µM → 50 µM					
Rab3A$_{Y84F}$:Rep15<br>500 µM → 50 µM	0.74 ± 0.02	11.1 ± 0.81 x 10^{-6}	-6.75	-3.20	-3.55

N.A. denotes not applicable

Supplementary References

1. Jumper, J. *et al*. Highly accurate protein structure prediction with AlphaFold. *Nature* **596**, 583-589 (2021).
2. Sarai, N., Kagawa, W., Kurumizaka, H. & Yokoyama, S. Crystal Structure of Holliday Junction Resolvase ST1444. (RIKEN Structural Genomics/Proteomics Initiative (RSGI), 2007).
3. Laskowski, R.A., Jablonska, J., Pravda, L., Varekova, R.S. & Thornton, J.M. PDBsum: Structural summaries of PDB entries. *Protein Sci* **27**, 129-134 (2018).
4. Middleton, C., Parker, J., Knott, G., White, M. & Bond, C. Crystal 'Unengineering': Reducing the Crystallisability of Sulfolobus solfataricus Hjc. *Australian Journal of Chemistry* **12**, 1818-1823 (2014).
5. Sievers, F. *et al*. Fast, scalable generation of high-quality protein multiple sequence alignments using Clustal Omega. *Mol Syst Biol* **7**, 539 (2011).
6. Ostermeier, C. & Brunger, A.T. Structural basis of Rab effector specificity: crystal structure of the small G protein Rab3A complexed with the effector domain of rabphilin-3A. *Cell* **96**, 363-74 (1999).
7. Kukimoto-Niino, M. *et al*. Structural basis for the exclusive specificity of Slac2-a/melanophilin for the Rab27 GTPases. *Structure* **16**, 1478-90 (2008).
8. Chavas, L.M. et al. Elucidation of Rab27 recruitment by its effectors: structure of Rab27a bound to Exophilin4/Slp2-a. *Structure* **16**, 1468-77 (2008).
9. Wu, M., Wang, T., Loh, E., Hong, W. & Song, H. Structural basis for recruitment of RILP by small GTPase Rab7. *EMBO J* **24**, 1491-501 (2005).
10. Rai, A. *et al*. bMERB domains are bivalent Rab8 family effectors evolved by gene duplication. *Elife* **5**(2016).
11. Rai, A., Bleimling, N., Vetter, I.R. & Goody, R.S. The mechanism of activation of the actin binding protein EHBP1 by Rab8 family members. *Nat Commun* **11**, 4187 (2020).
12. Lindsay, A.J. *et al*. Identification and characterization of multiple novel Rab-myosin Va interactions. *Mol Biol Cell* **24**, 3420-34 (2013).
