# Supplementary material for: Rep15 interacts with several Rab GTPases and has a distinct fold for a Rab effector
Source: Nat Commun. 2022 Jul 23;13:4262. doi: 10.1038/s41467-022-31831-1 (PMC9308819; doi:10.1038/s41467-022-31831-1)
Supplement: Supplementary file 3 — Reporting Summary [file 41467_2022_31831_MOESM3_ESM.pdf]

nature portfolio

Corresponding author(s): Amrita Rai and Roger Goody

Last updated by author(s): Jun 24, 2022

Reporting Summary

Nature Portfolio wishes to improve the reproducibility of the work that we publish. This form provides structure for consistency and transparency in reporting. For further information on Nature Portfolio policies, see our Editorial Policies and the Editorial Policy Checklist.

Statistics

For all statistical analyses, confirm that the following items are present in the figure legend, table legend, main text, or Methods section.

n/a	Confirmed	
☐	☒	The exact sample size (*n*) for each experimental group/condition, given as a discrete number and unit of measurement
☐	☒	A statement on whether measurements were taken from distinct samples or whether the same sample was measured repeatedly
☐	☒	The statistical test(s) used AND whether they are one- or two-sided<br>*Only common tests should be described solely by name; describe more complex techniques in the Methods section.*
☒	☐	A description of all covariates tested
☒	☐	A description of any assumptions or corrections, such as tests of normality and adjustment for multiple comparisons
☐	☒	A full description of the statistical parameters including central tendency (e.g. means) or other basic estimates (e.g. regression coefficient) AND variation (e.g. standard deviation) or associated estimates of uncertainty (e.g. confidence intervals)
☐	☒	For null hypothesis testing, the test statistic (e.g. *F*, *t*, *r*) with confidence intervals, effect sizes, degrees of freedom and *P* value noted<br>*Give P values as exact values whenever suitable.*
☒	☐	For Bayesian analysis, information on the choice of priors and Markov chain Monte Carlo settings
☒	☐	For hierarchical and complex designs, identification of the appropriate level for tests and full reporting of outcomes
☒	☐	Estimates of effect sizes (e.g. Cohen's *d*, Pearson's *r*), indicating how they were calculated

Our web collection on statistics for biologists contains articles on many of the points above.

Software and code

Policy information about availability of computer code

Data collection	X-ray diffraction data were collected on synchrotron beamline X10SA at the Swiss Light Source (Paul Scherrer Institute, Villigen, Switzerland).
Data analysis	X-ray datasets were processed and reduced with XDS package. Initial phasing was done by molecular replacement with PHASER and the atomic models were built with COOT (0.8.9.2)/Phenix AutoBuild and refined in PHENIX (1.20_4487). Structural figures were generated using PyMol (2.2.2 or 2.5.2). Isothermal titration calorimetry data analysis was done using the MicroCal software (Origin 7). All other data were analyzed with Origin9. Additional software including Imagej 1.51s and Adobe Illustrator CS4 (14.0.0) or Adobe Illustrator Artwork 26.0 were used.<br><br>The number of the colonies in each dish was counted using open available software open CFU (http://opencfu.sourceforge.net). For statistical analysis, the Student’s t-test (two-tailed) was applied.<br><br>Bioinformatics: Clustal Omega, PDBePISA server , DALI server.

For manuscripts utilizing custom algorithms or software that are central to the research but not yet described in published literature, software must be made available to editors and reviewers. We strongly encourage code deposition in a community repository (e.g. GitHub). See the Nature Portfolio guidelines for submitting code & software for further information.

Data

Policy information about availability of data

All manuscripts must include a data availability statement. This statement should provide the following information, where applicable:

- Accession codes, unique identifiers, or web links for publicly available datasets
- A description of any restrictions on data availability
- For clinical datasets or third party data, please ensure that the statement adheres to our policy

The plasmids and cell lines created in this study are available from the corresponding author upon reasonable request. The coordinates and structure factors generated in this study have been deposited in the Protein Data Bank (https://www.rcsb.org) and are available under the accession codes:
PDB 8A4A [https://www.rcsb.org/structure/unreleased/8A4A] (Rep15:Rab3C_Q222H_10-227)
PDB 8A4C [https://www.rcsb.org/structure/unreleased/8A4C] (Rep15:Rab3B)
PDB 8A4B [https://www.rcsb.org/structure/unreleased/8A4B] (Rep15 ΔN1: Rab3B_Q81L_18-190)

The previously published structures used in this study are available as PDB accession codes: 3RAB [https://www.rcsb.org/structure/3RAB], 2EO0 [https://www.rcsb.org/structure/2EO0], 1ZBD [https://www.rcsb.org/structure/1ZBD], 4TKD [https://www.rcsb.org/structure/4TKD], 2ZET [https://www.rcsb.org/structure/2ZET], 3BC1 [https://www.rcsb.org/structure/3BC1], 1YHN [https://www.rcsb.org/structure/1YHN], 5SZI [https://www.rcsb.org/structure/5SZI], 5LPN [https://www.rcsb.org/structure/5LPN], 6ZSI [https://www.rcsb.org/structure/6ZSI], respectively. AlphaFold structural prediction of Rep15 is available from the AlphaFold Protein Structure Database [https://alphafold.ebi.ac.uk/entry/Q6BDI9].

The sequences used in this study were obtained from Uniprot and the accession codes are described in Source Data file. Source data are provided in Source Data file. A reporting summary for this article is available as a Supplementary Information file.

Field-specific reporting

Please select the one below that is the best fit for your research. If you are not sure, read the appropriate sections before making your selection.

☒ Life sciences ☐ Behavioural & social sciences ☐ Ecological, evolutionary & environmental sciences

For a reference copy of the document with all sections, see nature.com/documents/nr-reporting-summary-flat.pdf

Life sciences study design

All studies must disclose on these points even when the disclosure is negative.

Sample size	Sample size was chosen based on the standard practices in the field (Rai et al., eLife2016, Rai et al., Nat Commun 2020). No statistical methods were used to predetermine the sample size.
Data exclusions	No data was excluded.
Replication	All biochemical and cellular assays are repeated at least three times with similar results, unless otherwise stated. The number of replicate for each experiment is shown in the figure legends and method. All replication attempts were successful.
Randomization	Randomization was applied during structure refinement where a random subset of test reflections (5%) was used during cross validation. No other randomization was done in the study.
Blinding	Blinding was not possible since data collection and analysis was performed by the same investigator.

Reporting for specific materials, systems and methods

We require information from authors about some types of materials, experimental systems and methods used in many studies. Here, indicate whether each material, system or method listed is relevant to your study. If you are not sure if a list item applies to your research, read the appropriate section before selecting a response.

Materials & experimental systems

n/a	Involved in the study	
☐	☒	Antibodies
☐	☒	Eukaryotic cell lines
☒	☐	Palaeontology and archaeology
☒	☐	Animals and other organisms
☒	☐	Human research participants
☒	☐	Clinical data
☒	☐	Dual use research of concern

Methods

n/a	Involved in the study	
☒	☐	ChIP-seq
☒	☐	Flow cytometry
☒	☐	MRI-based neuroimaging

Antibodies

Antibodies used	Primary antibodies used in this work:<br>1: ANTI-FLAG® antibody produced in rabbit F7425 (polyclonal, Sigma).<br>2: Anti-alpha-Tubulin monoclonal (DM1A) mouse IgG1 T9026 (Sigma).<br>Secondary antibodies used in this work:<br>1: IRDye® 800CW Goat anti-Rabbit IgG Secondary Antibody (LI-COR Biosciences).<br>2: IRDye® 680RD Goat anti-Mouse IgG Secondary Antibody (LI-COR Biosciences).
Validation	https://www.sigmaaldrich.com/DE/en/product/sigma/f7425 (Antibody dilution 1:1000)<br>https://www.sigmaaldrich.com/DE/de/product/sigma/t9026 (Antibody dilution 1:3000)<br>https://www.licor.com/bio/reagents/irdye-800cw-goat-anti-rabbit-igg-secondary-antibody (Antibody dilution 1:10000)<br>https://www.licor.com/bio/reagents/irdye-680rd-goat-anti-mouse-igg-secondary-antibody (Antibody dilution 1:10000)

Eukaryotic cell lines

Policy information about cell lines

Cell line source(s)	COS-7 (ATCC® CRL-1651™) and U138MG (ATCC: HTB-16TM)
Authentication	Cell lines were not authenticated.
Mycoplasma contamination	No mycoplasma contamination was detected for U138MG cells. COS-7 cells are not tested for mycoplasma contamination.
Commonly misidentified lines<br>(See ICLAC register)	No commonly misidentified cell lines were used.
